# Akt1 inhibition promotes breast cancer metastasis through EGFR-mediated β-catenin nuclear accumulation

**DOI:** 10.1186/s12964-018-0295-1

**Published:** 2018-11-16

**Authors:** Wei Li, Jiu-Zhou Hou, Jie Niu, Zhuo-Qing Xi, Chang Ma, Hua Sun, Chao-Jie Wang, Dong Fang, Qin Li, Song-Qiang Xie

**Affiliations:** 1 Institute for innovative drug design and evaluation, School of Pharmacy, Henan University, N. Jinming Ave, Kaifeng, 475004 China; 2 The Key Laboratory of Natural Medicine and Immuno-Engineering, Henan University, N. Jinming Ave, Kaifeng, 475004 China; 3 Institute of Chemical Biology, School of Pharmacy, Henan University, N. Jinming Ave, Kaifeng, 475004 China

**Keywords:** Akt1, EGFR, β-Catenin, PIKfyve, Metastasis

## Abstract

**Background:**

Knockdown of Akt1 promotes Epithelial-to-Mesenchymal Transition in breast cancer cells. However, the mechanisms are not completely understood.

**Methods:**

Western blotting, immunofluorescence, luciferase assay, real time PCR, ELISA and Matrigel invasion assay were used to investigate how Akt1 inhibition promotes breast cancer cell invasion in vitro. Mouse model of lung metastasis was used to measure in vivo efficacy of Akt inhibitor MK2206 and its combination with Gefitinib.

**Results:**

Knockdown of Akt1 stimulated β-catenin nuclear accumulation, resulting in breast cancer cell invasion. β-catenin nuclear accumulation induced by Akt1 inhibition depended on the prolonged activation of EGFR signaling pathway in breast cancer cells. Mechanistic experiments documented that knockdown of Akt1 inactivates PIKfyve via dephosphorylating of PIKfyve at Ser^318^ site, resulting in a decreased degradation of EGFR signaling pathway. Inhibition of Akt1 using MK2206 could induce an increase in the expression of EGFR and β-catenin in breast cancer cells. In addition, MK2206 at a low dosage enhance breast cancer metastasis in a mouse model of lung metastasis, while an inhibitor of EGFR tyrosine kinase Gefitinib could potentially suppress breast cancer metastasis induced by Akt1 inhibition.

**Conclusion:**

EGFR-mediated β-catenin nuclear accumulation is critical for Akt1 inhibition-induced breast cancer metastasis.

## Background

Breast cancer is the most common cancer in women and the second leading cause of female cancer death worldwide because of distant metastasis [1]. Numerous studies have shown that abnormal activation of the Akt signaling pathway promotes tumorigenesis by enhancing cancer cell survival, growth in breast cancer [2, 3]. Thus, a number of small-molecule inhibitors targeting Akt have been developed to test their activities against breast cancer in clinical trials [4, 5]. However, accumulating evidences from several laboratories revealed that Akt isoforms exhibit distinct functions in cancer progression in spite of their high sequence and structural homology [6–8].

The serine/threonine kinase Akt1, one of the three isoforms in the Akt family, has emerged as a suppressor of tumor metastasis in breast cancer [9, 10]. For example, Akt1 activation accelerates cell proliferation but inhibits cell motility and invasion in breast cancer cells, whereas Akt1 inhibition promotes Epithelial-to-Mesenchymal Transition in breast cancer [11–13]. However, the mechanism and downstream signals by which Akt1 inhibition regulates each step of breast cancer metastasis are not completely understood.

β-catenin is a major component of cell-cell adhesion structures and functions as a controller of cell migration, colony formation and stem cell properties through translocation into nucleus [14, 15]. Aberrant β-catenin accumulation in the cytoplasm usually translocates to the nucleus and was associated with tumor relapse and metastasis in breast cancer patients [16]. A study by Tzeng HE found inhibition of PI3K (phosphatidyl inositol 3-kinase) significantly enhanced the nuclear translocation of β-catenin in breast cancer cells [17]. Recently, Gao F et al. found endothelial Akt1 loss promotes prostate cancer metastasis via nuclear translocation of β-catenin [18]. Therefore, we concerned about whether β-catenin nuclear accmulation as an alternative pathway was responsible for breast cancer metastasis induced by Akt1 inhibition.

In this study, we discovered that knockdown of Akt1 induced β-catenin nuclear accumulation in breast cancer cells, while inhibition of β-catenin nuclear accumulation using XAV-939 could reverse Akt1 knockdown-induced breast cancer invasion.

## Materials and methods

### Reagents and antibodies

RPMI 1640 and fetal bovine serum (FBS) were purchased from Gibco (Grand Island, NY, USA). Dimethylsulfoxide (DMSO), Hoechst 33342, XAV-939, Gefitinib and YM201636 were purchased from Sigma (St. Louis, MO, USA). U0126 was purchased from Cell Signaling Technology (Beverly, MA, USA). Polyclonal anti-human β-catenin antibody, monoclonal anti-human EGFR antibody, monoclonal anti-human phospho-EGFR (Y^1068^) antibody, monoclonal anti-human β-actin antibody and the corresponding horseradish peroxidase-conjugated second antibodies were purchased from Santa Cruz Biotechnologies (Santa Cruz, CA, USA). Monoclonal anti-human EEA.1, monoclonal anti-human phospho-ERK_1/2_ (Thr^202^) antibody, polyclonal anti-human ERK_1/2_ and monoclonal anti-human Lamin B antibody were purchased from Cell Signaling Technology (Beverly, MA, USA). The secondary anti-mouse or anti-rabbit antibodies conjugated with Alexa Fluor 488 or Alexa Fluor 568 was purchased from Invitrogen (Carlsbad, CA, USA). Two different Akt1 specific siRNAs purchased from GE Dharmacon (Lafayette, CO, USA) were used: ACA AGG ACG GGC ACA TTAA (1^#^siRNA), CAA GGG CAC TTT CGG CAAG (2^#^siRNA).

### Cell culture and RNA interference

All cell lines used in this study were purchased from the Cell Bank of the Chinese Academy of Science (Shanghai, China). These cells were cultured in the RPMI1640 medium supplemented with 10% fetal bovine serum (FBS) at 37 °C in a humidified incubator containing 5% CO2. RNA interference was performed using Lipofectamine® RNAiMAX (Life Technologies) according to the manufacturer’s instructions. 24 h after transfection, cells were collected.

### Luciferase assay

MCF-7 and MDA-MB-231 cells at 80% confluence were co-transfected with myr-Akt1 or siRNA, TCF-driven TOPflash reporter plasmid (Millipore) (400 ng) and control Renilla luciferase (25 ng) using 1.5 μl of Lipofectamine 2000 (Life Technologies). After 24 h of co-culture, the transfected cells were lysed and the supernatant was collected for dual luciferase activity measurements (Promega, Madison, WI). Luciferase activity was normalized for transfection efficiency and graphed as ratio of TOPflash/FOPflash activity.

### Matrigel invasion assay

2 × 10^5^ cells were seeded on top of 8 μm chamber coated with a Matrigel (Corning, Bedford, MA, USA), 600 μL RPMI 1640 supplemented with 10% FBS were placed in the lower compartment. The inhibitors used in this study were added to both the top and bottom chambers of the Transwell. After incubation for 24 h, cells were fixed with 4% paraformaldehyde, stained with 0.1% crystal violet. The number of invading cells was determined by counting five high-power fields (× 40) randomly on each membrane.

### Quantitative real-time polymerase chain reaction assay

Total RNA of breast cancer cells was extracted using TRIzol (Invitrogen, Carlsbad, CA, USA) according to the manufacturer’s instructions. Extracted RNA (1 μg) was used for reverse transcription with MMLV reverse transcriptase (Takara, Tokyo, Japan). Quantitative real-time PCR was carried out using SYBR Premix Ex Taq™ II (Takara, Japan) and the amplification conditions consisted of an initial incubation at 95 °C for 10 min, followed by 40 cycles of 95 °C for 10 s and 60 °C for 30 s. The results were analyzed using comparative threshold cycle (Ct) method for relative quantification. Glyceraldehyde phosphate dehydrogenase (GAPDH) was used as internal control.

### Enzyme-linked immunosorbent assay (ELISA)

Levels of human EGF, HB-EGF, TGF-α, β-Cellulin and amphiregulin in the cell culture supernatant were measured using ELISA kits (R&D Systems, Minneapolis, MN) in accordance with the instructions provided by the manufacturer. Absorbance was measured at 450 nm by V_max_ Kinetic microplate reader (Molecular Devices, Sunnyvale, CA).

### Cell fraction and Western blot

The total proteins were isolated from cancer cell lines using RIPA buffer. The nuclear proteins were isolated from cancer cell lines using Nuclear and Cytoplasmic Protein Extraction Kit (Beyotime, Shanghai, China) following the manufacturer’s instructions. Equal amounts of proteins were separated using 10% SDS-PAGE, and then transferred onto PVDF membranes. Afer blocking in 5% nonfat milk in PBS, the membranes were incubated with the indicated primary and secondary antibodies and detected by using the ECL plus reagents (Beyotime, Shanghai, China).

### Co-immunoprecipitation

Breast cancer cells transfected with plasmids were homogenized for 1 h in ice-cold lysis buffer containing 20 mM Tris-HCl (pH 7.4), 100 mM NaCl, 1% NP40, and complete protease inhibitor cocktail. The homogenates were then centrifuged at 12000 rpm for 10 min to yield the total protein extract in the supernatant. Protein extracts were then incubated with anti-Akt1 antibody at 4 °C for 3 h. Protein A/G agarose (Santa Cruz Biotechnology) was added to the samples, and the incubation was continued for another 12 h. Subsequently, the beads were washed 6 times with lysis buffer and boiled with SDS loading buffer at 100 °C for 5 min, then subjected to SDS-PAGE.

### Immunofluorescence assay

Cells were fixed for 10 min with 4% paraformaldehyde at room temperature, blocked with 5% BSA for 1 h at room temperature and stained overnight with primary antibody for β-catenin or EGFR and EEA.1 at 4 °C. Then cells were incubated for 1 h with appropriate secondary antibody. The nuclei were then stained with Hoechst 33342, images were captured by confocal fluorescent microscope.

### Measurement of phosphatidylinositol-5-phosphate (PI5P) in cells

Breast cancer cells were plated at 1 million cells per 10 cm plate and labeled with 10 μCi/mL 3H-myo-inositol for 48 h. At 48 h, the cells were transfected with siRNA, After 4 h, the medium was replaced with normal 1640 medium supplemented with 10% FBS and cells were allowed to grow for another 24 h. Phosphoinositides including PI5P were extracted, deactylated and separated by high performance liquid chromatography as previous described [19].

### Mouse model of lung metastasis

All animal procedures were performed with the approval of the Institutional Animal Care and Use Committee at Henan University. Tumor metastasis assays were performed using an intravenous breast cancer mouse model as previous described [11]. Briefly, 4 T1 cells (1 × 10^5^) were injected into the lateral tail vein of Balb/c mice. To ensure all mice bore actively growing lung tumors before the drug treatment, pulmonary metastasis was allowed to develop for 7 days. On day 8, a low dose of MK-2206 (60 mg/kg) or/and Gefitinib (200 mg/kg) was administered orally once daily, three times per week for two weeks. Then the mice were sacrificed and lungs were removed. After fixed with 4% paraformaldehyde for 1 day, lung metastases nodules were counted.

### Statistical analysis

Statistical analyses were performed with GraphPad Prism 5 for Windows (GraphPad Software, La Jolla, CA). All data are expressed as mean ± SEM. For normalized data analysis, data was confirmed that normality assumption was satisfied and analyzed using paired sample t-test (dependent t-test) and/or further confirmed with non-parametric test Wilcoxon signed rank test. For all other analyses, Student’s two-tailed t-test or ANOVA test were used to determine significant differences between treatment and control values. Differences with *P* < 0.05 were considered statistically significant.

## Results

### β-Catenin nuclear accumulation contributes to Akt1 inhibition-mediated breast cancer metastasis

**Fig. 1:**
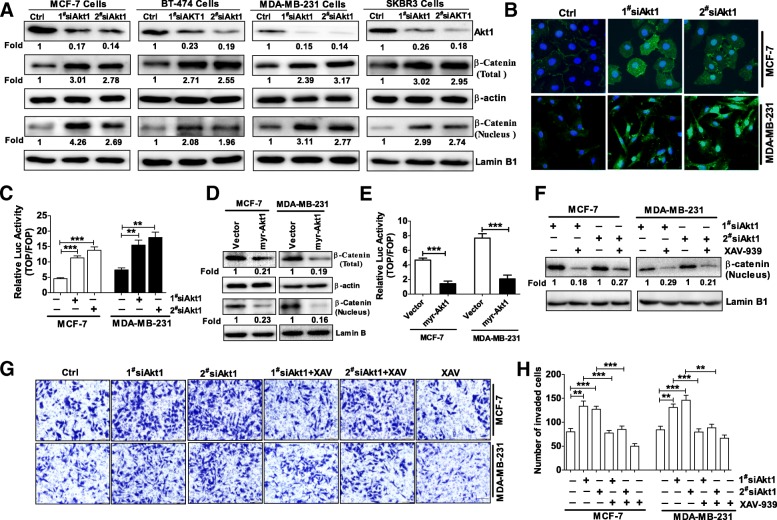
Akt1 inhibition promoted breast cancer cell invasion through inducing β-catenin nuclear accumulation. (**a**) Western blot of Akt1, β-catenin total and nuclear protein expression in MCF-7, BT-474, MDA-MB-231 and SKBR3 cells after treated with two Akt1 siRNA. β-actin and Lamin B1 were used as an internal control respectively. Note that Akt1 siRNA could knockdown the protein expression of Akt1, but upregulate the expression of β-catenin total and nuclear protein in these four distinct breast cancer cells, *n* = 5 per group. (**b**) Immunohistochemical staining of β-catenin (Green) in MCF-7 and MDA-MB-231 cells after treated with two Akt1 siRNA. Cell nuclei were stained with Hoechst 33342 (Blue). Note that Akt1 siRNA could induce nuclear β-catenin accumulation in MCF-7 and MDA-MB-231 cells, *n* = 3 per group. (**c**) Akt1 siRNA enhanced the β-catenin-dependent transcriptional activity. The firefly luciferase activity of each sample was normalized to the Renilla luciferase activity. **p < 0.01, ***p < 0.001 compared with Control group, one-way ANOVA, *n* = 5 per group. (**d**) Expression of myr-Akt1 in MCF-7 and MDA-MB-231 cells reduced the expression of β-catenin total protein as well as its nuclear accumulation. (**e**) Expression of myr-Akt1 in MCF-7 and MDA-MB-231 cells reduced the β-catenin-dependent transcriptional activity. The firefly luciferase activity of each sample was normalized to the Renilla luciferase activity. ***p < 0.001 compared with Vector group, A two-tailed unpaired t-test, *n* = 5 per group. (**f**) Western blot analysis showing that the AXIN stabilizer XAV939 could reverse Akt1 siRNA induced β-catenin nuclear accumulation, *n* = 5 per group. (**g**-**h**) Transwell assay with Matrigel demonstrated that Akt1 knockdown could enhance the invasion ability of MCF-7 and MDA-MB-231 cells, while XAV-939 reversed Akt1 siRNA-induced breast cancer invasion. **p < 0.01, ***p < 0.001, one-way ANOVA, *n* = 5 per group

In order to explore the mechanisms by which Akt1 inhibition promotes breast cancer metastasis, we first used two specific siRNA to knockdown Akt1 in four distinct breast cancer cell lines including MCF-7 (ER^+^, PR^+^, HER2^−^, wild type EGFR, wild type PTEN, wild type p53), BT-474 (ER^+^, PR^+^, HER2^+^, wild type EGFR, wild type PTEN, mutant p53), MDA-MB-231 (ER^−^, PR^−^, HER2^−^, wild type EGFR, wild type PTEN, mutant p53)and SKBR3 (ER^−^、PR^−^、HER2^+^, wild type EGFR, wild type PTEN, mutant p53) cells. As expected, the protein expression of Akt1 was downregulated in these breast cancer cells after treated with 20 nM Akt1 siRNA for 24 h (Fig. 1a). In addition, the expression of β-catenin total protein was upregulated when Akt1 was knocked down in these breast cancer cells (Fig. 1a). Multiple studies suggested aberrant β-catenin accumulation in the cytoplasm usually translocates to the nucleus where it acts as a transcriptional co-activator to activate a series of genes that are associated with cell migration and invasion [15, 20]. Hence, we further detected the expression of β-catenin nuclear protein in breast cancer cells after Akt1 knockdown. Using Western blot assay, we found the nuclear protein expression of β-catenin was upregulated in these cells treated with Akt1 siRNA (Fig. 1a). In order to confirm these results, we then select MCF-7 and MDA-MB-231 cells to perform immunofluorescence staining. As shown in Fig. 1b, MCF-7 and MDA-MB-231 cells displayed strong β-catenin staining in the cytoplasm and nucleus after Akt1 knockdown (Fig. 1b). Nuclear-localized β-catenin usually interacts with transcription factors of the T cell factor (TCF) family and promotes the target gene expression [21, 22]. Therefore, the TOP/FOP Flash reporter assay was employed to examine β-catenin transcriptional activity in MCF-7 and MDA-MB-231 breast cancer cells. As the results shown MCF-7 and MDA-MB-231 cells treated with Akt1 specific siRNAs displayed higher β-catenin transcriptional activity compared with control group (Fig. 1c). Then, we expressed constitutively activated, myristoylated Akt1 (myr-Akt1) or empty vector in MCF-7 and MDA-MB-231 cells to investigate the expression of β-catenin, the transfection efficiency of myr-Akt1 is above 70% in MCF-7 and MDA-MB-231 cells. As shown in Fig. 1d, active Akt1 significantly reduced the total protein expression of β-catenin as well as its nuclear accumulation. The TOP/FOP Flash reporter assay also suggested β-catenin transcriptional activity was downregulated in MCF-7 and MDA-MB-231 cells expressing myr-Akt1(Fig. 1e). Collectively, these results indicated Akt1 inhibition promoted the expression of β-catenin as well as its nuclear accumulation. To study whether β-catenin nuclear accumulation contributes to Akt1 inhibition induced breast cancer cell invasion, the Axin stabilizer XAV-939 was used in the following study. As a stabilizer of Axin, XAV-939 promotes the degradation of β-catenin, thus leading to decreased β-catenin nuclear translocation [23, 24]. In line with these reports, we found that knockdown of Akt1 induced β-catenin nuclear accumulation was reversed by XAV-939 in MCF-7 and MDA-MB-231 cells (Fig. 1f). Meanwhile, we found the ability of tumor cell invasion increased dramatically after treated with Akt1 specific siRNA in MCF-7 and MDA-MB-231 breast cancer cells using transwell assay with Matrigel, while XAV-939 could reverse the enhanced invasive ability of breast cancer cells induced by Akt1 knockdown (Fig. 1g-h). Together, these results indicated that β-catenin nuclear accumulation contributes to Akt1 inhibition-mediated breast cancer cell invasion.

### Activation of EGFR contributes to β-catenin nuclear accumulation induced by Akt1 knockdown in breast cancer cells

**Fig. 2:**
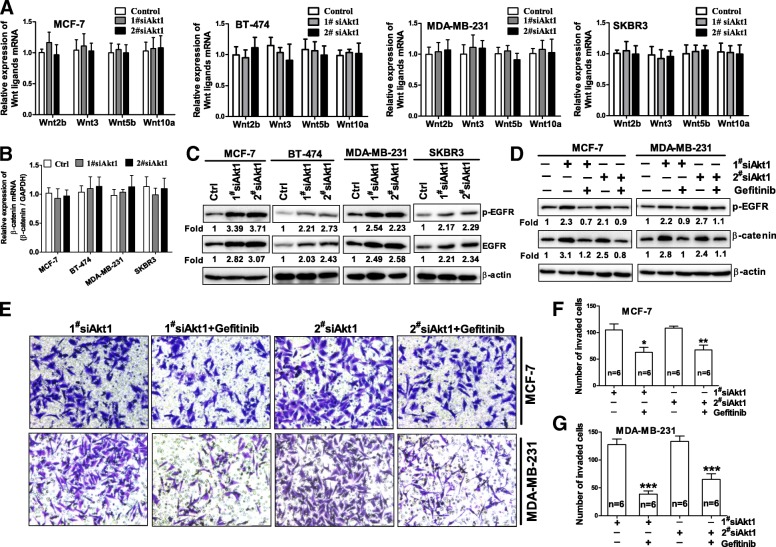
Activation of EGFR mediates β-catenin nuclear accumulation in Akt1-impaired MCF-7 and MDA-MB-231 cells. (**a**) Real time PCR analysis of Wnt2b, Wnt3, Wnt5b and Wnt10a mRNA expression in MCF-7, BT-474, MDA-MB-231 and SKBR3 cells after treated with Akt1 siRNA. Note that no significant alteration is observed on Wnt2b, Wnt3, Wnt5b and Wnt10a mRNA expression after treated with Akt1 siRNA for 24 h in these four distinct breast cancer cells. (**b**) Real time PCR analysis of β-catenin mRNA expression in MCF-7, BT-474, MDA-MB-231 and SKBR3 cells after treated with Akt1 siRNA. Note that no significant alteration is observed on β-catenin mRNA expression after treated with Akt1 siRNA for 24 h in these breast cancer cells. (**c**) Western blot analysis showed that knockdown of Akt1 enhanced both the phosphorylation at Tyr^1068^ and the total protein expression of EGFR in MCF-7, BT-474, MDA-MB-231 and SKBR3 cells, *n* = 5 per group. (**d**) Western blot analysis showed that Akt1 siRNA-induced β-catenin nuclear accumulation was almost completely blocked by Gefitinib (20 μM) after 24 h co-incubation in MCF-7 and MDA-MB-231 cells, *n* = 5 per group. (**e**-**g**) Transwell assay with Matrigel demonstrated that Gefitinib reversed AKT1 siRNA-induced breast cancer invasion in MCF-7 (**e**, **f**) and MDA-MB-231 cells (**e**, **g**). *p < 0.05, **p < 0.01, ***p < 0.001, one-way ANOVA, *n* = 5 per group

It has been reported inhibition of PI3K significantly enhanced the nuclear translocation of β-catenin through promoting Wnt ligands (including Wnt2b, Wnt3, Wnt5b and Wnt10a) expression in MDA-MB-231 cells [17]. Considering that Akt is one of the dominant downstream effector of PI3K signaling [25, 26], we asked whether aberrant expression of Wnt ligands contributed to β-catenin nuclear accumulation in MCF-7, BT-474, MDA-MB-231 and SKBR3 cells treated with Akt1 siRNA. Unexpected, we did not observe the upregulation of Wnt2b, Wnt3, Wnt5b and Wnt10a mRNA expression in these Akt1 siRNA treated cells (Fig. 2a). Obviously, Akt1 inhibition induced β-catenin nuclear accumulation was not caused by Wnt pathway activation in breast cancer cells. On the other hand, the mRNA expression of β-catenin in MCF-7, BT-474, MDA-MB-231 and SKBR3 cells treated with Akt1 siRNA was not changed compared with the control group (Fig. 2b), indicating the upregulated protein expression of β-catenin was not dependent on transcriptional regulation.

Numerous studies have demonstrated activation of EGFR usually stabilized and enhanced β-catenin nuclear accumulation [27, 28], this raises the possibility that β-catenin nuclear accumulation in Akt1-imparied cells may be induced by the activation of EGFR. To test the hypothesis, we first examined whether Akt1 inhibition could induce EGFR activation in breast cancer cells. As expected, our results shown that the phosphorylation levels of EGFR at Tyr^1068^ which is an indicator of EGFR activation was increased significantly in MCF-7, BT-474, MDA-MB-231 and SKBR3 cells (Fig. 2c). In addition, an increase in EGFR total protein expression was also observed in these Akt1 knockdown breast cancer cells (Fig. 2c). These results implied that the activation of EGFR may contribute to β-catenin nuclear accumulation in breast cancer cells.

In order to further confirm that β-catenin nuclear accumulation was stimulated by EGFR signal in Akt1-impaired cells, we tested the effect of Gefitinib, an inhibitor of EGFR tyrosine kinase on the β-catenin nuclear accumulation in the breast cancer cells treated with Akt1 siRNA. Using Western blot assay we found that Akt1 knockdown-induced β-catenin nuclear accumulation was almost completely blocked by Gefitinib (20 μM for 24 h) in MCF-7 and MDA-MB-231 cells (Fig. 2d). On the other hand, we also found Akt1 siRNA-induced the increase in the cell number invaded through Matrigel could be dramatically attenuated by Gefitinib in MCF-7 and MDA-MB-231 cells (Fig. 2e-g). Collectively, these results suggested the activation of EGFR mediates β-catenin nuclear accumulation in Akt1-impaired breast cancer cells.

### Knockdown of Akt1 induced the sustained activation of EGFR through inactivating PIKfyve in breast cancer cells

**Fig. 3:**
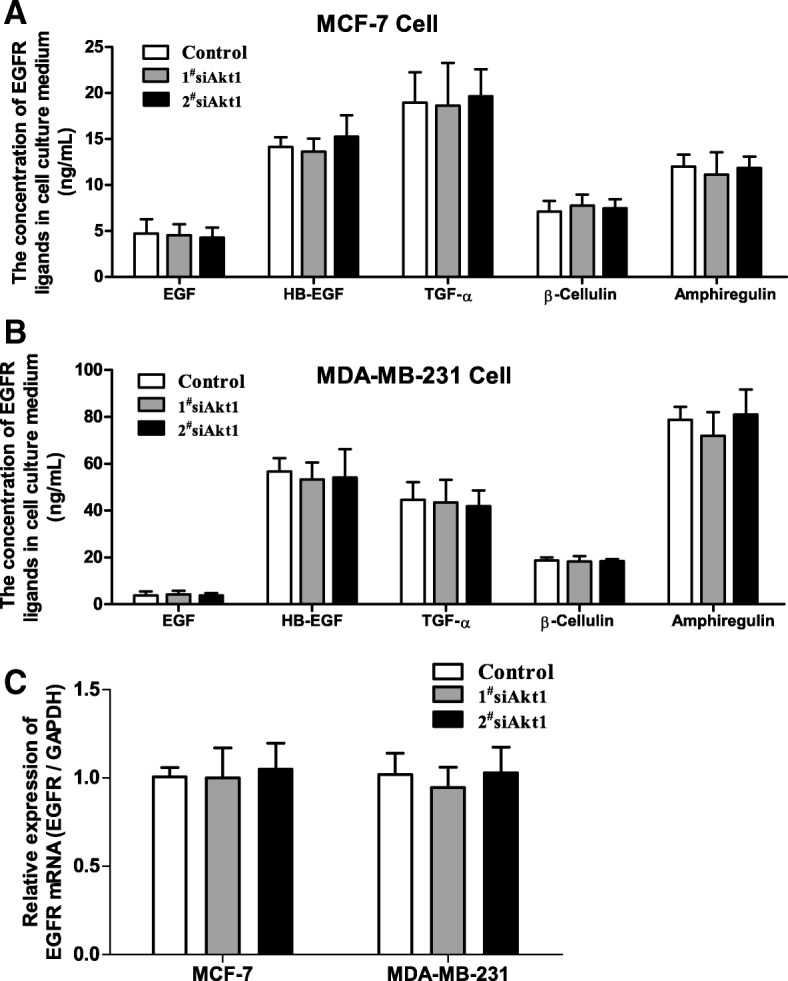
Knockdown of Akt1 could not promote the secretion of EGFR ligands and the mRNA expression of EGFR in MCF-7 and MDA-MB-231 cells. (**a**, **b**) The EGFR ligands EGF, HB-EGF, TGF-α, β-Cellulin and amphiregulin was detected in the cell culture medium using ELISA assay. Note that no significant alteration was observed on the secretion of EGF, HB-EGF, TGF-α, β-Cellulin and amphiregulin in the cells transfected with Akt1 siRNA, one-way ANOVA, n = 5 per group. (**c**) Real time PCR analysis showed that the expression of EGFR mRNA in MCF-7 and MDA-MB-231 cells after treated with Akt1 siRNA. Note that no significant alteration is observed on EGFR mRNA expression after treated with Akt1 siRNA for 24 h in MCF-7 and MDA-MB-231 cells, one-way ANOVA, *n* = 5 per group

As mentioned above, knockdown of Akt1 induced sustained activation of EGFR in breast cancer cells. This raises the possibility that the activation of EGFR may be induced by its ligands including epithelial growth factor (EGF), Heparin-binding epidermal growth factor-like growth factor (HB-EGF), transforming growth factor α (TGF-α), β-Cellulin and amphiregulin in Akt1-impaired cells. To test our speculation, the cell culture medium was harvested and analyzed by ELISA assay. Unexpected, no significant alteration was observed on the secretion of EGF, HB-EGF, TGF-α, β-Cellulin and amphiregulin in MCF-7 and MDA-MB-231 cells transfected with AKT1 siRNA compared with the control group (Fig. 3a-b). These results implied that the activation of EGFR in Akt1 impaired breast cancer cells occurred independently of its ligands binding. Then, we wonder whether the increased in EGFR total protein expression in Akt1 impaired breast cancer cells was dependent on its transcriptional regulation. To test this notion, we detected the mRNA expression of EGFR using real-time PCR. However, the results revealed that knockdown of Akt1 in MCF-7 and MDA-MB-231 cells failed to promote the mRNA expression of EGFR (Fig. 3c), suggesting that additional target is contributing to the overexpression of EGFR in Akt1 impaired breast cancer cells.

**Fig. 4:**
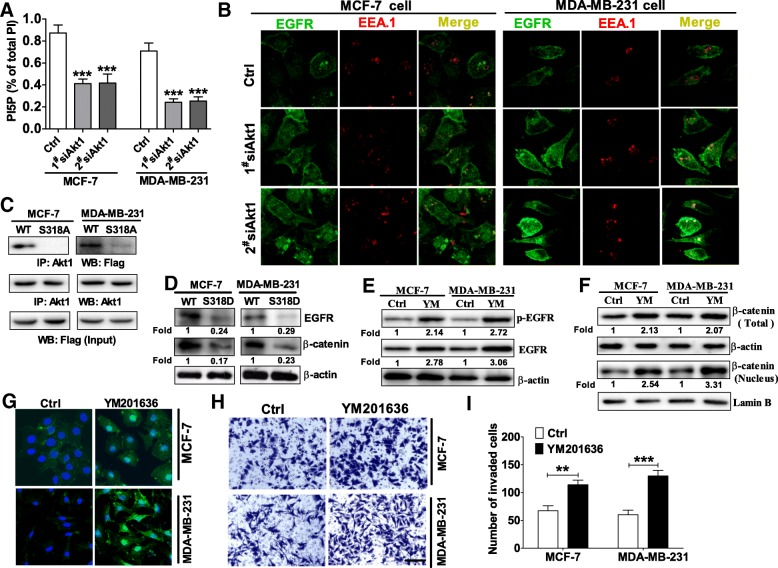
Knockdown of Akt1 promoted EGFR activation by dephosphorylating and inactivating PIKfyve. (**a**) knockdown of Akt1 decreased PI5P production in MCF-7 and MDA-MB-231 cells, PI: Phosphoinositides, ***p < 0.001, one-way ANOVA, *n* = 5 per group. (**b**) Knockdown of Akt1 induced an increased EGFR co-localization with the early endosomes marker EEA.1 in MCF-7 and MDA-MB-231 cells. (**c**) Co-immunoprecipitation assay was performed to determine the phosphorylation status of PIKfyve by Akt1. Note that Flag-PIKfyve WT but not a S318A mutant PIKfyve binds to Akt1 in MCF-7 and MDA-MB-231 cells, *n* = 5 per group. (**d**) Expression of a phosphorylation-mimic PIKfyve S318D mutant in breast cancer cells could induce a lower expression of EGFR and β-catenin protein than did WT PIKfyve in MCF-7 and MDA-MB-231 cells, *n* = 3 per group. (**e**) Western blot analysis showed that inhibition of PIKfyve with YM201636 enhanced the phosphorylation and total protein expression of EGFR in MCF-7 and MDA-MB-231 cells, n = 5 per group. (**f**) Western blot analysis showed that inhibition of PIKfyve with YM201636 could upregulate the expression of β-catenin total and nuclear protein in MCF-7 and MDA-MB-231 cells, *n* = 5 per group. (**g**) Immunofluorescence staining showed PIKfyve inhibitor YM201636 could induce β-catenin nuclear accumulation in MCF-7 and MDA-MB-231 cells. (**h**-**i**) Transwell assay with Matrigel demonstrated that PIKfyve inhibitor YM201636 enhanced the ability of invasion in MCF-7 and MDA-MB-231 cells. H: representative of images of breast cancer cells; I: analysis of the invade cells. **p < 0.01, ***p < 0.001, two-tailed unpaired t-test, n = 5 per group

Er EE et al. have found inhibition of Akt1 reduces the degradation of EGFR through inactivating PIKfyve, which correlates with an increase in the localization of EGFR in early endosomes in MCF-10A cells [29]. Therefore, we determined whether the increased phosphorylation levels of EGFR at Tyr^1068^ and EGFR total protein in Akt1 impaired breast cancer cells was due to the reduction in degradation through inactivating PIKfyve. To test this hypothesis, we first examined the concentration of phosphatidylinositol-5-phosphate (PI5P) which was used to reflect PIKfyve activity in previous studies [29, 30]. As Fig. 4a shown, knockdown of Akt1 decreased PI5P production in MCF-7 and MDA-MB-231 cells, implying that the activity of PIKfyve was inhibited after Akt1 knockdown. As mentioned above, a decreased PIKfyve activity was associated with an increased in the localization of EGFR in the early endosomes. Indeed, an increased EGFR co-localization with the early endosomes marker EEA.1 could be detected in MCF-7 and MDA-MB-231 cells treated with Akt1 siRNA (Fig. 4b), suggesting that PIKfyve activity was inhibited when Akt1 was knocked down in breast cancer cells. It has been reported that Akt1 activates the phosphoinositide kinase activity of PIKfyve through phosphorylating PIKfyve at Ser^318^ site, therefore we transfected MCF-7 and MDA-MB-231 cells with WT Flag-PIKfyve or S318A mutant PIKfyve and performed co-immunoprecipitation assay. Our results suggested that Flag-PIKfyve WT but not a S318A mutant PIKfyve binds to Akt1, confirming that Akt1 phosphorylates PIKfyve at Ser^318^ site in breast cancer cells (Fig. 4c). Moreover, we also examined whether expression of a phosphorylation-mimic PIKfyve S318D mutant in breast cancer cells could induce EGFR degradation. As results shown in Fig. 4d, the phosphorylation-mimic PIKfyve S318D mutant had a lower level in EGFR and β-catenin protein expression than did WT PIKfyve in MCF-7 and MDA-MB-231 cells. To investigate whether inhibition of PIKfyve is required for β-catenin nuclear accumulation via the prolonged activation of EGFR in breast cancer cells, we then treated MCF-7 and MDA-MB-231 cells with a PIKfyve specific inhibitor YM201636. As illustrated in Fig. 4e, both phosphorylated and total EGFR expression was upregulated in MCF-7 and MDA-MB-231 cells treated with 200 nM YM201636 for 24 h. Meanwhile, we also observed an increased expression of β-catenin total and nuclear protein in MCF-7 and MDA-MB-231 cells incubated with 200 nM YM201636 for 24 h (Fig. 4f). Of course, a strong β-catenin staining located in the cytoplasm and nucleus was also observed in MCF-7 and MDA-MB-231 cells after treated with YM201636 (Fig. 4G). To test whether inhibition of PIKfyve effects breast cancer cell invasion, we then performed transwell assay with Matrigel. As shown in Fig. 4h-i, tumor cells incubated with 200 nM PIKfyve inhibitor YM201636 for 24 h displayed a higher ability of migration. Collectively, these data demonstrate that knockdown of Akt1 prolonged EGFR activation through inactivating PIKfyve in breast cancer cells.

### Akt1 inhibition led to more sustained ERK signaling in breast cancer cells

**Fig. 5:**
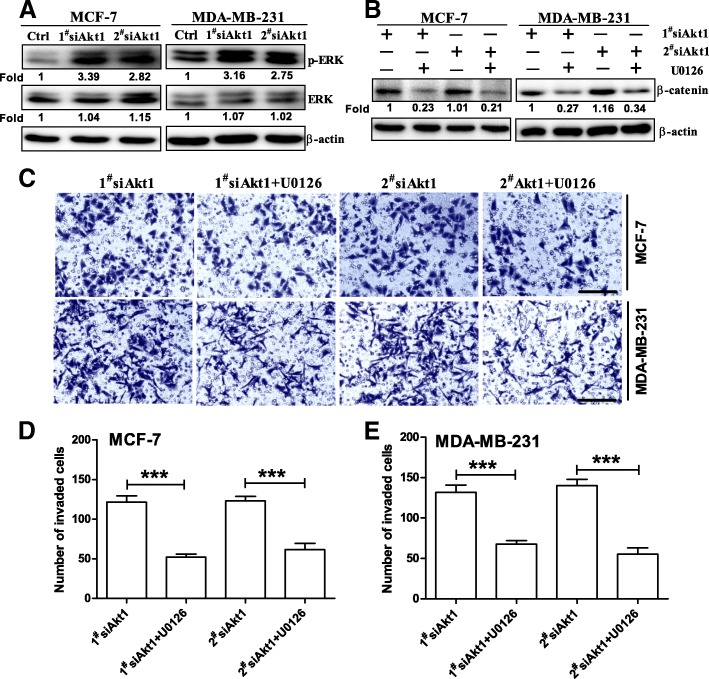
Knockdown of Akt1 induced the activation of ERK signaling in breast cancer cells, thus resulting in β-catenin nuclear accumulation and cancer cell invasion. (**a**) Western blot analysis showed that knockdown of Akt1 enhanced the activation of ERK signaling pathway in MCF-7 and MDA-MB-231 cells, n = 5 per group. (**b**) Western blot analysis showed that Akt1 siRNA-induced β-catenin nuclear accumulation was almost completely blocked by ERK inhibitor U0126 (20 μM) after 24 h co-incubation in MCF-7 and MDA-MB-231 cells, n = 5 per group. (**c**-**e**) Transwell assay with Matrigel demonstrated that ERK inhibitor U0126 (20 μM for 24 h) reversed Akt1 siRNA-induced breast cancer invasion in MCF-7 (**c**, **d**) and MDA-MB-231 cells (**c**, **e**). ***p < 0.001, one-way ANOVA, n = 5 per group

As described in previous report, EGFRs were accumulated in the early endosomes, resulting in prolonged activation of ERK in Akt-impaired MCF-10A cells [29]. Consistent with the findings, we discovered that Akt1 knockdown could significantly increase ERK phosphorylation in MCF-7 and MDA-MB-231 cells (Fig. 5a). Because previous studies have found activation of ERK was required in β-catenin nuclear accumulation, we hence explored whether ERK inhibitor U0126 could reverse β-catenin nuclear accumulation induced by Akt1 siRNA. As expected, we found that Akt1 siRNA-induced β-catenin nuclear accumulation was blocked by ERK inhibitor U0126 in MCF-7 and MDA-MB-231 cells (Fig. 5b). Subsequently, we also found ERK inhibitor U0126 could reverse AKT1-induced increase in the cell number invaded through Matrigel (Fig. 5c-e). Together, these data indicated that Akt1 inhibition led to increased activation of ERK signaling in breast cancer cells, resulting in β-catenin nuclear accumulation and cancer cell invasion.

### EGFR tyrosine kinase inhibitor Gefitinib blocked breast cancer metastasis induced by Akt1 inhibitor in vivo

**Fig. 6:**
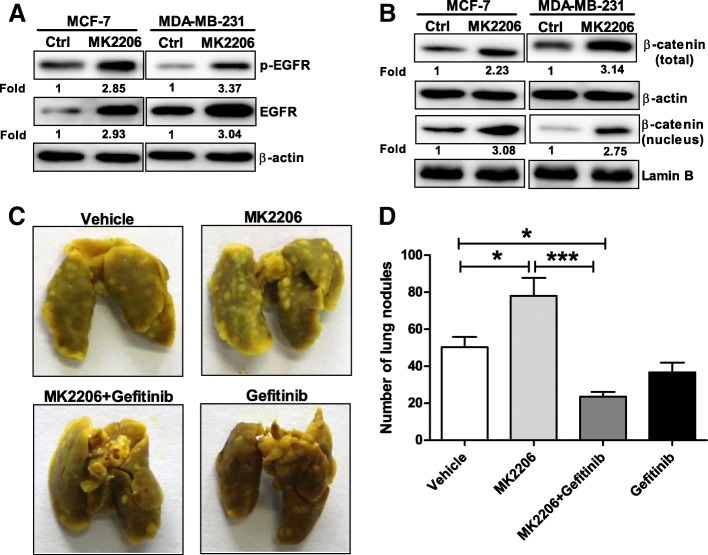
Inhibition of Akt1 with MK2206 induced breast cancer metastasis through EGFR-mediated β-catenin nuclear accumulation. (**a**) Inhibition of Akt1 with MK2206 promotes the protein expression of p-EGFR and EGFR in MCF-7 and MDA-MB-231 cells, n = 5 per group. (**b**) Inhibition of Akt1 with MK2206 induced an increase in the expression of β-catenin total and nuclear protein in MCF-7 and MDA-MB-231 cells, n = 5 per group. (**c**) Representative photograph of metastatic lung nodules. (**d**) Quantification of the lung nodules. Note that a low dose of MK-2206 significantly increased the numbers of tumor metastatic nodules in tumor-bearing mice, while Gefitinib significantly reduced the number of lung metastasis in mice that received MK-2206 administration. *p < 0.05, ***p < 0.001, one-way ANOVA, *n* = 10 per group

MK-2206 is a pan-Akt inhibitor, which has potential to induce EMT in breast cancer cells at a low dosage of 0.2 μM through inhibition of Akt1 [11]. Therefore, we asked whether MK-2206 enhance breast cancer metastasis via EGFR mediated β-catenin nuclear accumulation. Using Western blot assay, we found the expression of EGFR and β-catenin was upregulated in MCF-7 and MDA-MB-231 cells treated with 0.2 μM MK-2206 for 24 h (Fig. 6a-b). To evaluate the possibility that MK-2206 may induce metastatic potential when they are used in clinic for breast cancer therapy, we inoculated 4 T1 cells into the lateral tail vein of Balb/c mice to establish a mouse model of lung metastasis. Then a low dose of MK-2206 (60 mg/kg) was administered orally and the number of lung metastasis was counted. As shown in Fig. 6c-d, we found the Akt inhibitor MK2206 significantly increased the numbers of tumor metastatic nodules in tumor-bearing mice. Given that Akt1 inhibition promotes the activation of EGFR in breast cancer cells, we further examined whether the inhibitor of EGFR tyrosine kinase Gefitinib could suppress breast cancer metastasis induced by Akt1 inhibitor. Therefore, Gefitinib (200 mg/kg) were also administered orally prior to MK-2206 administration. The results showed Gefitinib significantly reduced the number of lung metastasis in mice that received MK-2206 administration (Fig. 6c-d). These results suggested Gefitinib may provide therapeutic benefits by limiting the metastatic potential when Akt1 inhibitor was used to treat breast cancer.

## Discussion

Accumulating reports suggest that overexpression of Akt1 in breast cancer cells blocks cell motility and invasion [31, 32]. This raised the possibility that the use of Akt inhibitors as anti-cancer agents may potentially promote breast cancer metastasis. Indeed, activation of Akt1 has been shown to accelerate tumorigenesis but suppresses tumor invasion in transgenic mouse models [32, 33]. Studies from the Brugge laboratory documented that Akt1 inhibits breast cancer cell motility through the suppression of ERK activation [34]. Recently, Li CW have found inhibition of Akt1 using MK-2206 induced epithelial-to-mesenchymal transition through blocking Twist1 degradation in breast cancer [11]. Consistent with these reports, our study also suggested knockdown of Akt1 promoted metastasis of breast cancer. Of course, the unexpected effects of Akt1 suppression on cancer metastasis are reported not only in breast cancer but also in prostate, liver, head & neck and non-small cell lung cancer cells (NSCLC) [11, 18, 35, 36]. Our results provided the evidence that EGFR-mediated β-catenin nuclear accumulation is critical for the Akt1 inhibition-induced breast cancer metastasis. In support of our results, Gao F et al. found Akt1 loss results in β-catenin translocation from the barrier junctions to the cytosol and nucleus in prostate cancer. Notably, they suggested increased production of TGF-β1 and its receptor TGF-β RII was responsible for β-catenin nuclear accumulation in prostate cancer. Moreover, they also observed that knockdown of Akt1 results in a reduction in β-catenin total protein expression in prostate cancer. In the case of breast cancer cells, knockdown of Akt1 induced an elevated expression of β-catenin total protein. We supposed that these conflicting results may be due to the different tumor types. In addition, we also suggested that knockdown of Akt1-induced β-catenin nuclear translocation was independent of the p53 status of cells, because the same results appeared in both the cells with wild type p53 and the cells with mutant p53.

It has been demonstrated that β-catenin expression was regulated by the PI3K/Akt signaling pathway in multiple cancer types such as hepatocellular carcinoma, renal cell carcinoma and colorectal cancer [37–39]. For example, when the PI3K/Akt signaling pathway was inhibited, β-catenin was degraded by adenomatosis polyposis coli polyprotein and its expression was reduced. In contrast, a recent study has shown the pan-PI3K inhibitor GDC-0941 significantly enhanced the nuclear translocation of β-catenin in breast cancer cell through stimulation of the transcription of Wnts [17]. However, we did not detect any changes of Wnt ligands in breast cancer cells treated with Akt1 siRNA. We supposed the discrepancies between these two studies may be due to the different protein kinases were inhibited in breast cancer, because some studies found many critical cellular processes are driven by PI3K-dependent but Akt-independent signaling to promote malignant phenotypes [40]. Besides Wnt ligands, EGFR signal stabilized and enhanced β-catenin nuclear accumulation by phosphorylated regulation [28]. Indeed, in Akt1 impaired breast cancer cells, we also documented EGFR mediates β-catenin nuclear accumulation. In support of our report, Timmermans-Sprang et al found inhibition of the PI3K/mTOR pathway in breast cancer was associated with enhanced expression of β-catenin and EGFR, implying that enhanced EGFR may function as a key signaling intermediate of β-catenin nuclear accumulation [41].

EGFR belongs to the ErbB family of receptor tyrosine kinases and was frequently overexpressed nearly in all subtypes of breast cancer patients [42]. Increased expression of EGFR in the primary tumor is associated with unregulated proliferation, malignant transformation, metastasis and resistance to apoptosis of cancer cells [43, 44]. EGFR carries out these functions through activation of Ras-Raf-MEK-ERK, PI3K-Akt-mTOR and Src-STAT3 pathways [45, 46]. However, inhibition of PI3K/Akt or MEK/ERK signaling pathway has been shown to induce the activation of multiple receptor tyrosine kinases (RTKs) that reactivate the pathway which attenuates its anti-tumor effects [41, 47]. The addition of RTK inhibitors can prevent this reactivation of RTKs and cause profound cell death and tumor regression [48–50]. Consistent with these reports, we also found that Akt1 inhibition induced the overexpression of EGFR contributing to metastasis in breast cancer in the present study. Interestingly, we note that the knockdown of Akt1 did not induce the upregulation of EGFR mRNA expression in breast cancer cells, suggesting that the upregulation of EGFR is a transcription independent regulation. Previous study found activated EGFR can be internalized in endosomes to be degraded via lysosome-mediated degradation pathway, while in Akt1-impaired cells EGFRs were unable to reach the lysosomal compartment for degradation, resulting in an increased protein abundance of EGFR in the early endosomes [29]. In support of this report, we did find more co-localization of EGFR with the early endosomes marker EEA.1 in Akt1-impaired breast cancer cells.

The phosphoinositide 5-kinase (PIKfyve) is a critical enzyme for the synthesis of PtdIns (3,5)P2 that has been implicated in both endosomal morphology and various membrane transport events. For example, Kim J et al. found PIKfyve is a direct mediator in the transport of EGFR from the cell surface through the cytoplasmic vesicular space to the nucleus in human bladder cancer cells [51]. Pharmacological inhibition of PIKfyve results in a block to the lysosomal degradation of EGFR in normal breast epithelial cell line [29]. PIKfyve was also proposed to be implicated in oncogenesis and cancer cell migration. For example, PIKfyve promotes cell migration and invasion through activation of Rac1 in lung carcinoma, osteosarcoma or rhabdomyosarcoma cells, while inhibition of PIKfyve resulted in a significant decrease in cell migration velocity and persistence [52]. Contrast with the report, our study suggested inhibition of PIKfyve activity promotes cell migration and invasion in breast cancer cells. We suppose this may because PIKfyve plays different roles in different cancers.

To the best of our knowledge, we disclosed for the first time that EGFR-mediated β-catenin nuclear accumulation is critical for the Akt1 inhibition-induced breast cancer metastasis. Of course, we did not exclude that the same signaling pathway present in other malignancies, because combined inhibition of EGFR and PI3K/Akt pathways could produce synergistic anti-tumor effects in lung cancers [53]. Given that inhibition of Akt1 enhanced breast cancer metastasis in mice, and many pan-AKT inhibitors are currently undergoing clinical trails for breast cancer treatment [5, 54], it is of particular importance to determine whether such inhibitors may induce metastasis in the treatment of breast cancer. If administration of these agents to patients have a promoting effect on the metastatic process, our findings indicated that the use of Akt inhibitor and Gefitinib at the same time may provide therapeutic benefits. Of course, further studies are necessary to determine the therapeutic benefit of combining Akt inhibitors and EGFR tyrosine kinase inhibitor in a clinical setting.

## Conclusion

We here disclose a novel Akt1/PIKfyve/EGFR/β-catenin signaling pathway, which contributes to the metastasis of breast cancer. Furthermore, the tyrosine kinase inhibitor of EGFR may provide therapeutic benefits by limiting the metastatic potential when Akt1 inhibitor was used to treat breast cancer.

## Abbreviations

EGFR: Epithelial growth factor receptor
ERK: Extracellular signal regulated kinase
HB-EGF: Heparin-binding epidermal growth factor-like growth factor
PI3K: Phosphatidyl inositol 3-kinase
PIKfyve: The phosphoinositide 5-kinase
TGF-α: Transforming growth factor α
